# The athletes heart—from acute stimulus to chronic adaptation

**DOI:** 10.1093/bmb/ldae021

**Published:** 2024-12-07

**Authors:** Joseph D Maxwell, David Oxborough

**Affiliations:** Research Institute of Sports and Exercise Science, Cardiovascular Health Science Research Group, Liverpool John Moores University, Liverpool, Tom Reilly Building, L3 3AF, United Kingdom; Liverpool Centre for Cardiovascular Science, University of Liverpool, Liverpool, Liverpool John Moores University and Liverpool Heart & Chest Hospital, Liverpool, United Kingdom; Cardio-Respiratory Unit, Liverpool University NHS Foundation Trust, Liverpool, Mount Vernon Street, L7 8XP, United Kingdom; Research Institute of Sports and Exercise Science, Cardiovascular Health Science Research Group, Liverpool John Moores University, Liverpool, Tom Reilly Building, L3 3AF, United Kingdom; Liverpool Centre for Cardiovascular Science, University of Liverpool, Liverpool, Liverpool John Moores University and Liverpool Heart & Chest Hospital, Liverpool, United Kingdom

**Keywords:** Athletes heart, exercise physiology, cardiac remodelling

## Abstract

**Introduction:**

The complex phenomenon of the athlete’s heart (AH) describes the chronic physiological structural and functional adaptation secondary to repeated exposure of an acute exercise stimulus.

**Sources of Data:**

This narrative review is based on published evidence.

**Areas of agreement:**

Highly trained athletic individuals frequently display cardiac parameters which are suggestive of an AH and can exceed the traditional ‘normal’ limits.

**Area of controversy:**

The physiological processes underpinning the extent of cardiac adaption and how this is closely linked to exercise type, but also sex, ethnicity, and body size.

**Growing points:**

Since its seminal description by Morganroth and colleagues in 1975, our understanding of the AH has evolved in tandem with improvements in cardiac imaging techniques alongside the exploration of more diverse athletic populations. This narrative review aims to provide a balanced discussion of the multi-factorial nature of structure and function of the AH with specific reference to the unique physiological exercise stimuli.

**Areas timely for developing research:**

Despite great interest in cardiac adaptations across a broad spectrum of athletic populations, future research designs should consider the use of new and novel imaging techniques to enhance our understanding of the acute cardiovascular responses which ultimately mediates such adaptations, especially in athletic populations underrepresented in the literature.

## Introduction

It is well established that regular exercise training is associated with a range of both structural and functional cardiac adaptions, a phenomenon often termed, *the Athlete’s Heart (AH)* [1]. Exercise induced adaptations are heterogenous, and can vary significantly due to sex, age, ethnicity, training status and training type [2–5]. Physiological adaptations to chronic exercise can occur across all cardiac chambers including increased left ventricular (LV) wall thickness, increased LV and right ventricular (RV) cavity sizes, atrial dilation, alongside reduced indices of resting systolic function (i.e. LV ejection fraction) when compared to non-athletes [5,6]. Indeed, it is such adaptations which make the assessment of the AH *vs* underlying pathology challenging in the clinical setting, thus highlighting the importance of understanding this interaction between chronic exercise training and cardiac adaptation [7].

The process of exercise induced cardiac remodeling, whereby cardiac chambers change in size, geometry, mass and function, is a physiological phenomenon [8]. This phenomenon is a crucial compensatory adaptation to the increased hemodynamic load applied to the heart during repeated acute exercise bouts. Indeed, when discussing factors that determine remodeling within the AH, it is essential to consider the magnitude of the hemodynamic stimulus to the heart as determined through exercise intensity and duration, often measured by indices such as metabolic equivalent (METS) [9].

**Figure 1:**
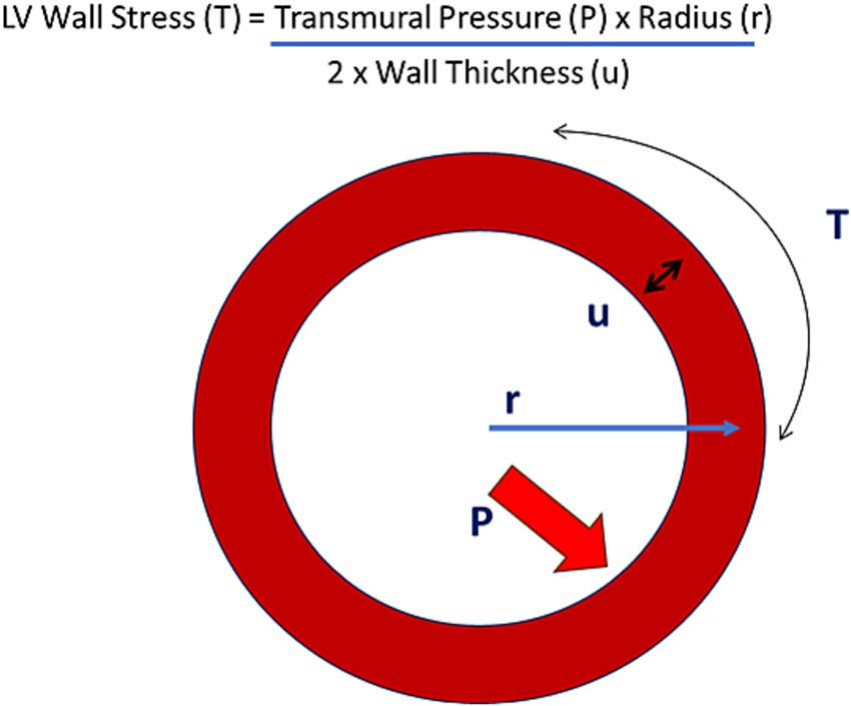
Adapted *La place* law of the heart.

The hemodynamic load exerted on the heart is dependent on the exercise type. For example, during acute aerobic exercise, increases in stroke volume (SV) are largely driven by enhanced venous return, leading to an increased LV volume (i.e. preload). This increase in preload and therefore chamber radius (i.e. end diastolic volume (EDV)) leads to increases in wall stress (according to the *La Place Law—*Fig. 1) and ultimately a greater magnitude of contractility (Frank-Starling mechanism). Alternatively, SV during isometric exercise (albeit intensity dependent) remains stable or even decreases, secondary to a decrease in preload and an increase in afterload [10]. Increases in blood pressure during isometric/resistance exercise can be hugely significant and far exceed expected pressures, with one landmark study showing systolic blood pressures (invasive brachial pressure) surpassing 320 mmHg during double leg press [11]. It is apparent that any adaptions to the geometry of the cardiac chambers may act as a physiological normalization to the variations in cardiac load [12].

**Figure 2:**
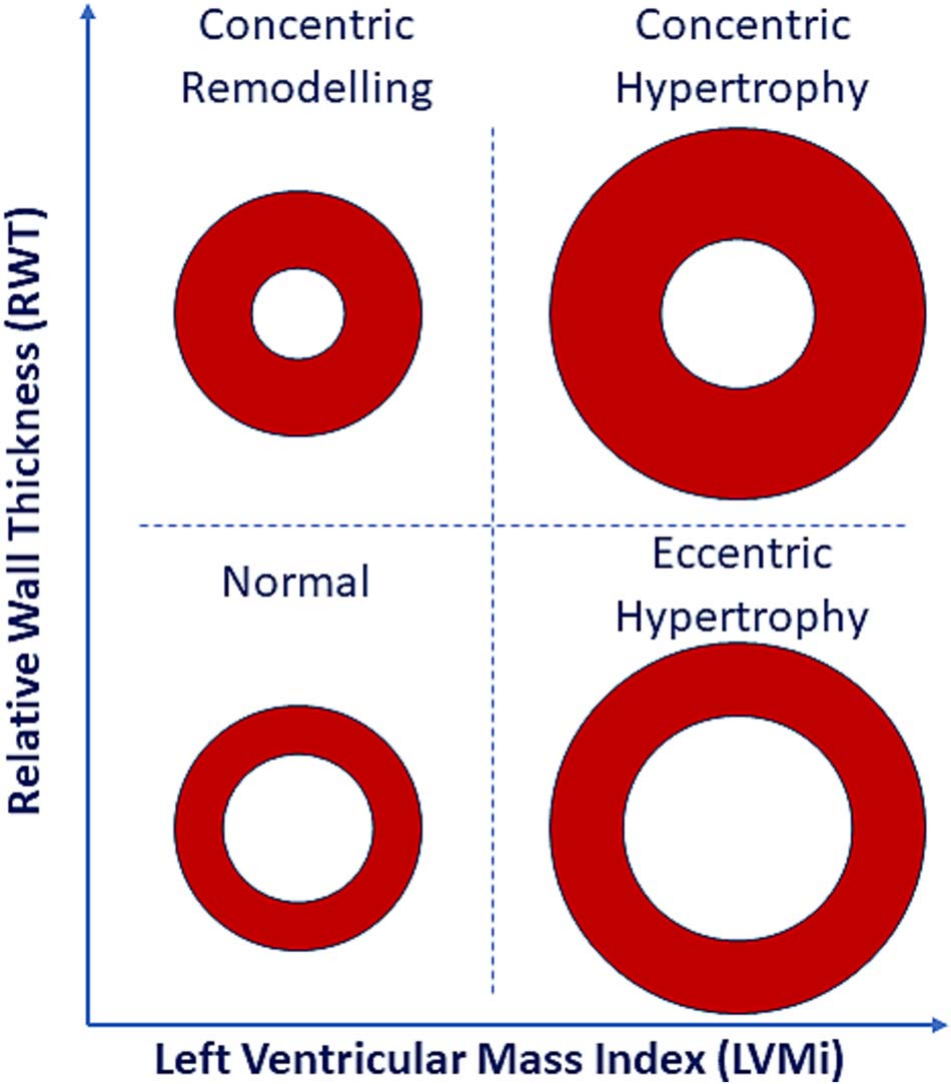
Simplified diagram representing the different forms of left ventricular geometry. Normal geometry; RWT = normal and LVMi = normal. Concentric remodeling; RWT = increased and LVMi = increased. Concentric hypertrophy; RWT = increased and LVMi = increased. Eccentric hypertrophy; RWT = normal and LVMi = increased. *Note:* Different cardiac imaging modalities and clinical guidelines use different criteria for increased RWT and LVMi.

The type of exercise training has historically been a focus of discussion with regards to cardiac adaptations, with original observations by Morganroth, et al. [13] suggesting that the geometry of the LV (LV mass indexed (LVMi) in relation to the relative wall thickness (RWT)) is largely dependent on the exercise training stimulus (Fig. 2). This seminal work from 1975 described how athletes from different sporting disciplines display an almost ‘exercise specific LV geometry’. In the subsequent years however, numerous studies have challenged the *Morgonroth Hypothesis* presentng evidence refuting the resistance aspect of the hypothesis due to the lack of concentric hypertrophy seen in strength trained athletes [14–16]. This evidence raises questions around the nature and impact of the specific stimuli on the heart during both resistance and endurance exercise. The growing AH evidence base has also increased our understanding of the multi-factorial nature of physiological cardiac remodeling. It is apparent that cardiac adaptation is dependent on other demographics including ethnicity, sex and body size. It is therefore, pertinent to summarize and explore the potential physiological mechanisms underpinning this heterogenous adaptation [7,17].

Physiological adaptation of biological systems and organs such as the heart allows for efficiency and is achieved through an associated relationship between structure and function. It is understandable that the morphological adaptations seen in the AH will occur alongside functional adaptation [7]. Increased chamber size (both ventricular and atrial), increased wall thickness and low resting function are frequently observed in athletic populations [5,18]. It is important to fully understand the physiological process which drive such adaptations to exercise, and factors which can influence these changes.

## Left ventricular adaptations

The LV has by far received the most attention when assessing the AH with many studies having attempted to quantify the magnitude of athletic adaptation and to directly align any remodeling with a specific exercise stimulus/discipline. The original description of this sport ‘specific’ cardiac adaptation came from Morganroth, et al. [13] who demonstrated, using echocardiography, that athletes who engage in regular isometric (resistance) exercise (e.g. wrestling and shotput) displayed normal LV end-diastolic volume (LVEDV) and increased wall thickness, whereas athletes who participate in isotonic (aerobic) sports (e.g. swimming and running) presented with increased LVEDV and normal wall thickness (Fig. 2). The working hypothesis for these dichotomous adaptations was based on the hemodynamic stimulus that the heart is exposed to during these exercise bouts, i.e. resistance training applies significant pressure overload (increased afterload) and endurance exercise applying significant volume overload (increased preload) [13]. Whether these divergent findings can solely be attributed to exercise stimulus is controversial [19], however knowledge of the fundamental acute exercise physiology can assist in our understanding of this phenomenon.

The Mitchell criteria refers to a classification which categorizes sporting disciplines according to the relative amount of static and dynamic mechanical action of the skeletal muscles which, although not originally intended, is often used to explain sport specific cardiac adaptations [20]. It is now well established that sports with the highest dynamic components (e.g. cycling, rowing, running) display the greatest extent of cardiac adaptations [9]. Endurance (dynamic) exercise involves sustained elevations in cardiac output (CO) with reduced or normal peripheral vascular resistance, whereas resistance (static) exercise induces intensive cyclic bouts of increased peripheral vascular resistance with small increases in CO. Previous echocardiography studies have shown that elite male cyclists have greater LVEDV, increased LVMi and reduced EF compared to non-athletes [4] with similar observations in recreational marathon runners [21]. Similarly, cardiac magnetic resonance (CMR) studies have shown increased LVMi, greater EDV and reduced RWT in recreational marathon runners, triathletes, and elite ultrarunners [22–24]. Functional adaptations also occur, with reductions in resting EF noted in endurance athletes as low as 12% below ‘normal’ [6]. This reduced EF is likely secondary to the increased EDV with lesser contractile force required to generate an adequate resting SV [7]. The chronic structural remodeling to endurance exercise is driven by cardiac myocyte elongation in response to an increased diastolic strain and the addition of sarcomeres in series [25]. This ultimately leads to LV dilation to enhance SV during exercise (via the Frank-Starling mechanism), as the SV of highly trained athletes at maximum capacity can exceed 200mL, as compared to ~ 100-130 mL in untrained individuals [26].

The evidence and nature of cardiac remodeling secondary to resistance training is less consistent, with conflicting data. This uncertainty is somewhat surprising given that the adapted *La Place’s Law* states (Fig. 1) that LV wall stress (tension) is the product of transmural pressure (difference between intracavity and intrathoracic pressures) and the LV geometry [27,28], and it is reasonable to assume that this form of exercise induces significant cardiac hemodynamic loads. *A* review of LV geometry in resistance trained athletes identified that 37.5% of studies showed normal geometry, 37.5% showed concentric hypertrophy, and 25% of studies observed eccentric hypertrophy, causing speculation that such divergent findings could possibly be related to the ‘type’ of resistance trained athletes (i.e. powerlifts vs bodybuilders) [29]. Furthermore, a meta-analysis by Utomi, et al. [30] utilizing both CMR and echocardiography data, concluded that both endurance and resistance trained athletes demonstrate larger LV structures than sedentary controls (with greater dimensions in endurance athletes), yet minimal difference in wall thickness, challenging the traditional *Morganroth hypothesis.* Additionally, numerous studies using different imaging modalities have shown negligible adaptation to both short and long-term resistance training, especially when compared to endurance trained athletes [31–33]. One feasible explanation for this ‘enhanced’ adaptation in endurance athletes compared to resistance may relate to training duration. Given that the adaptation is largely attributed to hemodynamic load exerted on the heart, it is highly likely that athletes who train for longer durations (i.e. endurance/aerobic), thus have a higher cumulative hemodynamic cardiac stress, will show more profound remodeling [20]. The nature of resistance exercise, which involves cyclical bouts of effort (~ < 10 s) interspersed with longer periods of rest, likely represents a significantly smaller hemodynamic challenge to the heart compared to an aerobic session in terms of cumulative duration. A further factor to consider when assessing the structure and function of the (more typically) resistance trained AH, is the use of image and performance enhancing drugs (IPEDS). Left ventricular hypertrophy and reduced LV systolic function have previously been reported in current IPED users compared to non-users [34,35], which can result in changes in overall cardiac geometry. Therefore, it is essential that researchers consider the role of IPEDs (both current and past users) when investigating any exercise specific adaptations.

It is important to acknowledge hemodynamic changes that may occur during and immediately following a Valsalva maneuver. Performing a brief Valsalva during intensive (~ > 80% maximal voluntary contraction) resistance exercise is largely unavoidable [36] and mediates an increase in intrathoracic pressure, leading to a reduction in preload and an acute increase in systemic blood pressure. It is logical to hypothesize that a marked increase in blood pressure and thus afterload would translate to significant increases in LV wall stress. However, a landmark study by Haykowsky, et al. [37] demonstrated that despite substantial increases in blood pressure, LV wall stress remained unchanged from baseline up to 1 repetition maximum on leg press. The concomitant increase in transmural pressure (increase in intrathoracic pressure) and LV geometry (reduced LVEDV and increases LV contractility) associated with Valsalva provides an explanation for the minimal change in wall stress observed [29]. Therefore, what was once deemed the ‘likely’ physiological process underpinning adaption to resistance exercise is in fact absent. Indeed, it is likely that it is these acute cardiovascular responses which explains, in part, the ‘reduced’ LV adaptation seen in resistance athletes compared to their endurance counterparts.

Defining the nature of the AH is further complicated when investigating sports which involve mixed training types (endurance and resistance). A recent meta-analysis of the AH in endurance athletes concluded that LV structure is significantly altered by endurance exercise. However, endurance sports that involved mixed training types (e.g. rowing) induced the greatest LV wall thickness changes [38].

While exercise type, intensity, and duration have typically formed the basis for understanding the AH, there are several other key parameters to consider. Historically, Caucasian athletes were the primary focus of research however a growing body of research has highlighted ethnicity as an important determinant in cardiac adaptation [39]. In a study directly comparing national level African/Afro-Caribbean athletes with age, sport and size matched control Caucasian athletes, the African/Afro-Caribbean athletes displayed increased LV wall thickness [40]. Additionally, African amateur endurance athletes have been shown to have a greater LVMi compared to Caucasian athletes, alongside an increased systolic blood pressure response at all stages of an incremental exercise test [41]. A higher systemic vascular resistance during exercise in African athletes [42] has been observed, mediating increases in afterload and hence providing a feasible explanation for increased LV wall thickness in African athletes (similar to that observed in hypertensive heart disease). A study by Riding et al. [43] utilizing a sample of 1222 athletes outlined that the prevalence of concentric hypertrophy and concentric remodeling was significantly higher in the African athlete population, although there was significant geographical variation within that cohort. Fundamentally, this largely consistent observation makes the screening for certain pathologies (i.e. Hypertrophic Cardiomyopathy) more challenging in the African athlete population.

With regards to other ethnicities, there is a scarcity of data on the AH. Elite level Arabic athletes have been shown to have significantly smaller LVEDV, LV wall thickness and LVMi compared to African and Caucasian athletes [44]. Japanese ultrarunners were found to have a mean LV end-diastolic diameter of 61.7 mm indicating a degree of dilation [45], however this study did present LV wall thickness’ up to 19 mm, which does raise suspicion of potential underlying cardiomyopathy. Nevertheless, the majority of the evidence does seem to suggest that other ethnicities appear to show similar structural and functional adaptations to that of the more frequently examined Caucasian athlete, indicating that the hemodynamic stimulus of the exercise is a primary factor [39,46].

Studies examining the AH have typically centered on the male athlete, however evidence demonstrates that highly trained females exhibit a similar adaptation patten to male athletes, albeit to a lesser magnitude [1]. For example, work by Rowland and Roti [47] comparing male and female adaption in competitive cyclists with similar training history, showed that males displayed a more profound increase in LV mass and LVEDV, even when adjusted for fat free body mass and body surface area. Furthermore, a study assessing sex differences across different sporting types (n = 1083) concluded that, whilst most female athletes display normal geometry, in dynamic sports, concentric hypertrophy/remodeling occurs in around 15% of males, however females tend to predominantly develop eccentric hypertrophy [48]. This data supports previous work stating that females have smaller LV dimensions and wall thicknesses compared to males [49]. Such findings suggest that concentric hypertrophy/remodeling in the female athlete is rare. Recent work by Oxborough et al. [50] has highlighted the use of a sex-specific allometric indexing approach in order to produce a more size independent index of cardiac geometry. Underlying mechanisms explaining sex differences are speculative, but may relate to increased levels of testosterone and a higher density of myocardial testosterone receptors in males [51]. Additionally, there is some evidence to suggest that the difference could be explained by a different cardiac load, with males on average having a higher systolic blood pressure at rest and during exercise [52].

It is now well established that anthropometric features have a profound impact on the LV and all cardiac structures, with evidence indicating that body size accounts for ~50% of the variability of LV mass in highly trained athletes [53], thus highlighting the importance of scaling. Current scaling methods utilized (primarily body surface area by Dubois regression) carry limitations and was based on only nine cadaveric subjects [54]. Numerous studies have now proposed the use of fat free mass (FFM) as a more appropriate tool for indexing cardiac parameters, especially in the athletic population. Given that changes in LV mass occur proportionally to changes in FFM, this supports our understanding that LV adaptation is driven by increases in metabolically active tissue [55]. Indexing to FFM, therefore, appears to be a sensible approach, however the practicalities of applying this approach in a clinical scenario, would be challenging. FFM has been shown to be the only independent predictor of both LV volumes and LV mass in endurance athletes [56]. Similar findings were observed in elite level athletes (both static and dynamic exercise types), with FFM showing the strongest correlation to LV mass and wall thickness [57,58]. Interestingly, sex differences in LV dimensions disappeared when adjusted for FFM, further highlighting its importance when discussing sex-specific AH adaptations [57]. It is worth noting, that even at the ‘extreme’ end of anthropometric measurements in athletes, LV adaptation to exercise is proportional to body size [59]

## Right ventricle adaptations

Whilst it is understandable that the LV as the systemic chamber has received by far the most attention, in recent decades the importance of exercise associated adaptations of the RV have become more apparent. While exercise associated adaptations to the RV have been described as ‘similar’ to that of the LV, it is important to acknowledge that there are some important differences, underpinned by physiological states, that are unique to the right heart and the pulmonary circulation. Interestingly, from an athlete screening perspective, the RV represents a major diagnostic challenge, as 14–24% of sudden cardiac deaths in the athletic population have been attributed to arrhythmogenic right ventricular cardiomyopathy (associated with significant RV mal-adaptation) [7,17].

Structurally, a wealth of data suggests that endurance exercise mediates increases in RV cavity, inflow, outflow as well as overall volumes, whereas the resistance trained athlete displays RV structural parameters not to dissimilar to that of sedentary controls [5,60–65]. Additionally, endurance athletes have been shown to have greater RV wall thickness compared to both resistance trained athletes and sedentary controls [63]. Data on RV systolic function in athletes is, however, less clear cut, with some evidence indicating RV function is lower than the traditional ‘normal’ in endurance athletes [66,67], whereas other studies have identified no differences in functional parameters between sporting types [65,68,69]. Such differences in finding may be related to different imaging techniques, or specific cohorts examined. One fascinating study by Arbab-Zadeh et al. [70] identified that, in previously sedentary healthy individuals following one year of intensive endurance training, the RV adaptations follow a different time course to those observed in the LV. The LV initially responded with an increase in mass without changes in volumes, with volume changes occurring 6–9 months following the onset of training. The RV, however, adapted with a balanced mass-volume ratio across the training program [70].

One potential reason for the lack of adaption within resistance trained athletes may be related to exposure time to increases in RV wall stress. During endurance-based exercise, the RV can be exposed to increased pulmonary artery pressure (PAP) and wall stress for prolonged periods of times > 1 hour. However, the short and cyclical increases in CO (largely HR driven rather than SV) associated with resistance exercise may not be a great enough stimulus to induce any adaptations. From a hemodynamic perspective, the fact that resistance trained athletes have similar RV chamber dimensions to sedentary controls implies that there is limited volume overload [63].

During aerobic exercise, both the systemic circulation and the pulmonary circulation adapt to the increased SV by the same magnitude. As insignificant as this may seem, unlike the systemic circulation, the pulmonary circulation at rest operates in a high compliance, low resistance, low afterload state. Therefore, during the onset of aerobic exercise, the systemic circulation has the ability to adjust to the increase in SV via a significant reduction in vascular resistance mediated by vasodilation of skeletal muscle and cutaneous vascular beds, whereas the pulmonary circulation has a limited ability to decrease vascular resistance, thereby functions at a much higher PAP during exercise [71]. This relationship between CO and PAP termed the P/Q relationship, has consistently shown a ~ 1 mmHg increase in PAP for each liter increase in CO, highlighting the significant PAP rise in well-trained athletes who show a 5-7 times increase in CO. [72] Additionally, increases in CO during exercise on the left heart causes left atrial pressure to rise (to increase LV filling in the presence of shorter diastolic filling times). This rise in left atrial pressure induces a ‘back-flow pressure effect’, ultimately resulting in raised pulmonary capillary wedge pressure [73], and thus RV systolic pressure. Therefore, during aerobic exercise, the RV is exposed to a greater load and relative wall stress than the LV due to a disproportional afterload increase. Interestingly, the RV and the interventricular septum both appear to display a prominent exercise-induced cardiac fatigue following endurance-based exercise [74]. There is even a suggestion that exposure to these repeated bouts of increased PAP and thus RV wall stress, can lead to/promote exercise induced cardiomyopathies [71].

Data regarding acute RV responses to resistance exercise is extremely limited, likely due to the technical difficulties with imaging the RV and its complex anatomy. One study by Motoji et al. [75] using non-invasive imaging techniques identified that resistance exercise evoked a smaller increase in the RV longitudinal contraction, pulmonary capillary wedge pressure, mean PAP and overall CO, when compared to aerobic exercise. Such results, however, must be viewed with caution, especially given the relatively low load of weightlifting (2.5 kg women/5 kg men) applied in this study. Indeed, this highlights the need for further robust research assessing the acute RV effects of resistance exercise.

Similar to studies investigating LV adaptations, RV focused work has also largely fixated on Caucasian athletes. A study aimed at establishing RV adaptation in African/Afro-Caribbean athletes demonstrated that ethnicity had little impact on RV adaptation, and only accounted for 2.3% of the variation in Right Ventricular Outflow Tract (RVOT) size [76]. Similar findings were observed in an adolescent population [77]. Interestingly, studies which have explored sex differences in the AH have often overlooked the RV. An echocardiography study of Masters athletics athletes (highly trained competitive athletes > 35 years old) showed negligible differences in the RV fractional area change and longitudinal function, but a greater RV end diastolic area (indexed to BSA [body surface area]) in men [78]. Whereas a large study (n = 1016) outlined that, whilst absolute RV values tend to be larger in males, when indexed to BSA, females show larger dimensions [69].

## Atrial adaptions

During acute aerobic exercise the atria are also exposed to significant volume overloads secondary to increased SV. Not only does atrial volume rise during acute exercise via increased venous return, but atrial pressure also rises to mediate increases in LVEDV/RV end-diastolic volume in the presence of reduced diastolic filling times. Consequently, both atria undergo significant volume and pressure overload during a bout of dynamic exercise. Furthermore, there are functional atrial parameters that respond to mediate ventricular filling at higher heart rates. Atrial reservoir function (elastic ability of the atrial walls to stretch) increases to maintain the atrial-ventricular pressure gradient [79]. This is then further supported via the atrial booster (pump) function which increases during acute exercise, further contributing to increased preload. An important study by Wright et al. [80] outlined that the phasic (reservoir, conduit and booster) roles of the LA differ with different exercise intensities, with LV filling at moderate intensity (the higher intensity in this particular study) primarily mediated by enhanced LV diastolic reserve with a greater conduit LA contribution. Indeed, those studies investigating acute atrial mechanics have used dynamic/aerobic based exercise, and to the best of our knowledge, no study has assessed such cardiac parameters during static/resistance exercise.

Incidence of bi-atrial dilation in the endurance athletic population is high regardless of imaging modality [21,30,81–85] and as such, in the presence of normal LV/RV filling pressures, it is largely deemed a normal variant of the AH in high-dynamic sporting athletes [7]. Previous work has even shown significant correlations between LA volume indexed to BSA and overall aerobic capacity [83,86]. Data from studies looking at the atrial size of resistance trained athletes has proven less consistent, with some evidence of significant atrial dilation [87], whilst other studies found little difference compared to sedentary controls [30,65,88], especially when indexed to BSA [89]. Indeed, indexed measurements of atrial volumes are important to remove a significant amount of the perceived differences in atrial sizes between sexes and sporting disciplines [89]. Atrial arrhythmias, specifically atrial fibrillation (AF), represent by far the most common arrhythmia (although still relatively rare) to arise in the athletes [90]. Whereas the mechanisms underpinning the development of AF in athletes are complex and multifactorial, there is some suggestion that atrial dilation/remodeling may play a role. A meta-analysis has shown that younger athletes (<55 years) were at higher risk of AF development, alongside those who participated in endurance and mixed sporting types [91], however larger prospective studies are needed to better understand associations between exercise and AF with relation to sex, ethnicity, training type and volume.

## Conclusion and future directions

The AH represents a complex model of structural and functional adaptions. In this narrative review, we have highlighted and discussed a range of variables which have a significant impact on the type and extent of the adaptation. Importantly, we have described some of the key acute physiological responses to exercise which, when applied frequently over time, have a profound impact on the AH. It is clear to see from the ever-growing body of literature, that our understanding of the AH will continue to grow, with different athletic cohorts being examined and new imaging modalities utilized. Whilst this remains important to understand chronic adaptation, in-keeping with our current review, we stress the need for new and novel study designs which further aid our knowledge on these acute cardiovascular responses, with a particular interest in exercise types that have received little attention and in underrepresented athletic populations.

## Data Availability

Data sharing not applicable to this article as no new data were created or analysed.
